# Safety and efficacy of regional citrate anticoagulation in continuous venovenous hemodialysis in the presence of liver failure: the Liver Citrate Anticoagulation Threshold (L-CAT) observational study

**DOI:** 10.1186/s13054-015-1066-7

**Published:** 2015-09-29

**Authors:** Torsten Slowinski, Stanislao Morgera, Michael Joannidis, Thomas Henneberg, Reto Stocker, Elin Helset, Kirsti Andersson, Markus Wehner, Justyna Kozik-Jaromin, Sarah Brett, Julia Hasslacher, John F. Stover, Harm Peters, Hans-H. Neumayer, Detlef Kindgen-Milles

**Affiliations:** Department of Nephrology, University Hospital Charité, Campus Mitte (CCM), Charitéplatz 1, D-10117 Berlin, Germany; Divison of Intensive Care and Emergency Medicine, Department of Internal Medicine, Medical University Innsbruck, Innsbruck, Austria; Department of Visceral and Transplant Surgery, University Hospital Charité, CVK, Berlin, Germany; Surgical Intensive Care, University Hospital Zurich, Zurich, Switzerland; Department of Anesthesiology, Oslo University Hospital, Oslo, Norway; Department of Acute Medicine, Oslo University Hospital, Oslo, Norway; Department of Anesthesiology, Leipzig University Hospital, Leipzig, Germany; Clinical Research, Fresenius Medical Care, Bad Homburg, Germany; Department of Anesthesiology, University Hospital Duesseldorf, Heinrich-Heine-University, Duesseldorf, Germany

## Abstract

**Introduction:**

Regional citrate anticoagulation (RCA) for continuous renal replacement therapy is widely used in intensive care units (ICUs). However, concern exists about the safety of citrate in patients with liver failure (LF). The aim of this study was to evaluate safety and efficacy of RCA in ICU patients with varying degrees of impaired liver function.

**Methods:**

In a multicenter, prospective, observational study, 133 patients who were treated with RCA and continuous venovenous hemodialysis (RCA-CVVHD) were included. Endpoints for safety were severe acidosis or alkalosis (pH ≤7.2 or ≥7.55, respectively) and severe hypo- or hypercalcemia (ionized calcium ≤0.9 or ≥1.5 mmol/L, respectively) of any cause. The endpoint for efficacy was filter lifetime. For analysis, patients were stratified into three predefined liver function or LF groups according to their baseline serum bilirubin level (normal liver function ≤2 mg/dl, mild LF >2 to ≤7 mg/dl, severe LF >7 mg/dl).

**Results:**

We included 48 patients with normal liver function, 43 with mild LF, and 42 with severe LF. LF was predominantly due to ischemia (39 %) or multiple organ dysfunction syndrome (27 %). The frequency of safety endpoints in the three patient strata did not differ: severe alkalosis (normal liver function 2 %, mild LF 0 %, severe LF 5 %; *p* = 0.41), severe acidosis (normal liver function 13 %, mild LF 16 %, severe LF 14 %; *p* = 0.95), severe hypocalcemia (normal liver function 8 %, mild LF 14 %, severe LF 12 %; *p* = 0.70), and severe hypercalcemia (0 % in all strata). Only three patients showed signs of impaired citrate metabolism. Overall filter patency was 49 % at 72 h. After censoring for stop of the treatment due to non-clotting causes, estimated 72-h filter survival was 96 %.

**Conclusions:**

RCA-CVVHD can be safely used in patients with LF. The technique yields excellent filter patency and thus can be recommended as first-line anticoagulation for the majority of ICU patients.

**Trial registration:**

ISRCTN Registry identifier: ISRCTN92716512. Date assigned: 4 December 2008.

## Introduction

Regional citrate anticoagulation (RCA) has become a widely used technique in continuous renal replacement therapy (CRRT). It is suggested in actual guidelines even in the absence of an increased bleeding risk for patients not already receiving systemic anticoagulation and without contraindications for citrate [1]. Prolonged filter patency, together with the possibility of avoiding systemic anticoagulation, led to an increasing acceptance of the technique [2–8]. However, regardless of the RCA protocol used, a considerable amount of citrate is infused into the systemic circulation. This citrate enters the citric acid cycle and is metabolized to bicarbonate [9]. Most dialysis-based protocols take the bicarbonate generation from infused citrate into account and use lower bicarbonate concentrations in the dialysate [6]. On one hand, such protocols usually allow better control of the acid–base state [10]; on the other hand, the metabolism of citrate is essential to maintain sufficient delivery of bicarbonate. As a consequence, in such protocols, impaired citrate metabolism translates into metabolic acidosis.

Another important issue in RCA is homeostasis of ionized calcium (iCa). The calcium bound to citrate is released if citrate is metabolized. In cases of impaired citrate metabolism, iCa is not released and systemic iCa decreases [11].

Several clinical observations raised concerns about the efficiency of citrate metabolism in the presence of liver failure (LF) [12]. Data showing reduced clearance of citrate in liver dysfunction led to clinical practice of avoiding RCA in patients with LF [11]. However, though coagulation often is impaired in liver dysfunction [13], filter clotting nevertheless occurs frequently [14]. Therefore, patients with impaired liver function might particularly benefit from RCA by avoidance of filter clotting without increasing bleeding risk or interfering with hemostasis through systemic anticoagulation.

To test the hypothesis that continuous venovenous hemodialysis and regional citrate anticoagulation (RCA-CVVHD) can also be recommended for patients with LF, the safety and efficacy of RCA-CVVHD in patients with different degrees of liver dysfunction were prospectively investigated.

## Material and methods

This study was a multicenter, open, prospective, observational trial (ISRCTN Registry identifier: ISRCTN92716512). The study was performed in accordance with the Declaration of Helsinki, and the protocol was approved by all local ethics committees. The names of the ethical bodies that approved the study in the centers involved are listed in the Acknowledgments section. All consecutive patients treated with RCA-CVVHD between January 2008 and February 2010 were considered for study participation. They were included if written informed consent was provided by themselves or by their legal representatives until at least 40 patients in each group were reached. All participating centers were university hospitals. The types of intensive care units (ICUs) were general (Department of Nephrology, Charité CCM, Berlin, Germany; Department of Anesthesiology, Leipzig University Hospital, Germany), surgical (Department of Visceral and Transplant Surgery, Charité CVK, Berlin, Germany; Department of Anesthesiology, University Hospital Duesseldorf, Germany; Surgical Intensive Care, University Hospital Zurich, Switzerland; Oslo University Hospital, Norway [OUS]), and medical (Department of Internal Medicine I, Medical University Innsbruck, Austria; OUS).

All patients fulfilled the following inclusion criteria: age ≥18 years and prescription of RCA-CVVHD for renal replacement therapy. The exclusion criteria were former use of RCA within 72 h before the study start, concomitant participation in another clinical trial, and previous participation in the same study.

A total of 133 patients were included in the study. Patients were stratified into three groups according to their liver function level, as defined by total serum bilirubin: normal liver function with bilirubin ≤2 mg/dl, mild LF with bilirubin >2 and ≤7 mg/dl, and severe LF with bilirubin >7 mg/dl.

All patients were treated with RCA-CVVHD using the multiFiltrate device (Fresenius Medical Care, Bad Homburg, Germany). The treatment protocol published by Morgera et al. [6] with a calcium-free and bicarbonate-reduced dialysate (Ci-Ca Dialysate K2 Plus; Fresenius Medical Care) was used. The dialysate flow was initially set as indicated by clinical needs. The blood flow was set at three times the dialysate flow. For anticoagulation, 4 % trisodium citrate solution was infused into the arterial line of the extracorporeal circuit with an initial dose of 4 mmol/L blood. The citrate dose was stepwise adapted to achieve a target postfilter iCa range of 0.25–0.35 mmol/L. Calcium chloride solution was infused into the venous line of the extracorporeal circuit at an initial dose of 1.7 mmol/L effluent and was adjusted to keep iCa within the physiologic range of 1.12–1.20 mmol/L. The ratio of dialysate flow to blood flow was increased in stepwise fashion in cases of metabolic alkalosis and likewise decreased in cases of acidosis [10].

The observation period included the first 72 h of the RCA-CVVHD treatment, which corresponded to the time point of a regular filter exchange as specified by the manufacturer.

Blood samples for arterial and postfilter analysis of pH, blood gases, and electrolytes (iCa, potassium, sodium) were taken and analyzed immediately on a bedside automated analyzer. All other laboratory parameters were analyzed in the central laboratories of the participating centers (all accredited according to the International Laboratory Accreditation Cooperation).

All measurements of arterial iCa and postfilter iCa, as well as arterial pH and actual bicarbonate, within the first 72 h of treatment were considered for analysis. Measurements of other serum electrolytes and total serum protein were analyzed at baseline and after 12, 24, 48, and 72 h. Additional laboratory parameters were analyzed at baseline and at the end of the observation period.

Endpoints for safety evaluation were occurrence of severe alkalosis or acidosis (pH ≥7.55 or ≤7.20, respectively) and severe hyper- or hypocalcemia (iCa ≥1.5 or ≤0.9 mmol/L, respectively) of any cause. The endpoint for efficacy was filter lifetime.

### Statistics

Descriptive statistics are presented as mean ± standard deviation (SD) for continuous variables. For categorical variables, relative frequencies are shown if not stated otherwise. Individual data for treatment prescription and acid–base measurements were recorded as changes occurring during the study period. Graphics and descriptive summaries at specific time points are based on the individual last observation carried forward imputation method (prescription data) and cubic spline interpolation estimates (iCa and acid–base measurements) per patient. Filter survival analysis was based on individual filter data. For overall filter survival, observations were censored at the end of the observation period only; for clotting-free survival, observations were censored when the filter was stopped for any reason except clotting. Comparison between liver groups was performed using one-way analysis of variance, the Kruskal-Wallis test, and Fisher’s exact test where appropriate. Spearman’s rank correlation was used to assess relationships between continuous variables. Reasoning for sample size determination was based on the detection chance of rare events. The chosen sample size of 120 provides a 90 % probability that events with a general risk of 2 % or more would be detected, and within each liver group (n = 40) events with an occurrence probability of 5.5 % would have a 90 % chance of being observed. All analyses were performed with SAS version 9.2 software (SAS Institute, Cary, NC, USA).

## Results

### Baseline data

A total of 133 patients were included, of whom 48 were stratified into the normal liver function group, 43 into the mild LF group, and 42 into the severe LF group.

The type of ICU admission, the major reason for ICU admission, and the risk, injury, failure, loss of kidney function, end-stage kidney disease (RIFLE) classes at initiation of RCA-CVVHD were similar, with the exception of cardiac disorders, which were a more frequent reason for ICU admission in the groups with normal liver function and mild LF (42 % and 44 % vs. 19 % in severe LF; *p* = 0.02). Overall types of ICU admission were medical (56 %), post–cardiac surgery (14 %), post–other surgery (24 %), posttrauma (4 %), and other (2 %). Major causes for ICU admission were respiratory failure (42 %), sepsis (39 %), and circulatory failure (35 %). RIFLE classes were 13.5 % risk, 15.8 % injury, and 67.7 % failure. Three percent had preexisting dialysis-dependent chronic renal failure. The causes of liver dysfunction did not differ between the mild and severe LF groups (*p* = 0.16). The etiologies of LF were 39 % ischemic, 27 % multiple organ dysfunction syndrome (MODS), 10 % alcohol abuse, 6 % viral hepatitis, and 18 % unknown or other.

The most common reason (stated as percentage of patients, with multiple reasons possible) for choosing RCA was either risk (74 %) or presence (20 %) of bleeding. In 14 %, RCA was the standard anticoagulant. An additional indication for citrate anticoagulation (8 %) was heparin-induced thrombocytopenia type II. A total of 100 patients were treated with RCA-CVVHD de novo, and 33 patients were switched to RCA from other RRT modalities. The average duration of previous RRT sessions was 4.1 ± 4.8 days.

The patients’ characteristics at baseline are given in Table 1. Other laboratory parameters at baseline and at study end are given in Table 2. Of note, at baseline, the mean international normalized ratio (INR) did not differ significantly in the LF function groups (normal liver function 1.7 ± 1.0, mild LF 1.5 ± 0.3, severe LF 1.8 ± 0.7), and bilirubin and INR did not show a relevant correlation in the study population (Spearman’s ρ = 0.18).

**Table 1:** Patients’ baseline characteristics according to liver function groups

	Normal liver function (bilirubin ≤2 mg/dl)	Mild liver failure (bilirubin 2–7 mg/dl)	Severe liver failure (bilirubin ≥7 mg/dl)	*p* Value	Total
Number of patients	48	43	42		133
Age (yr)	70 ± 11	56 ± 15	61 ± 15	<0.0001	63 ± 15
Male sex	67 %	70 %	79 %	0.46	71 %
Body weight (kg)	79.9 ± 17.8	83.9 ± 25.0	78.1 ± 20.9	0.46	80.7 ± 21
Height (cm)	170.6 ± 12.5	171.5 ± 10.4	174.2 ± 7.2	0.31	172.0 ± 10
Length of ICU stay at study start (days)	2 ± 2	4 ± 9	7 ± 9	0.004	4 ± 8
SOFA score	12 ± 3	15 ± 3	16 ± 3	<0.001	14 ± 3
Diuresis (ml/day)	1006 ± 1335	620 ± 787^a^	603 ± 774^a^	0.11	754 ± 1027
Mechanical ventilation	79 %	74 %	71 %	0.70	75 %
FiO_2_ (L/L)	0.44 ± 0.17	0.48 ± 0.22	0.46 ± 0.22	0.72	0.46 ± 0.2
MAP (mmHg)	73 ± 12	74 ± 13	76 ± 12	0.61	74 ± 12
Vasopressor support	75 %	86 %	79 %	0.42	80 %

*FiO*_*2*_ fraction of inspired oxygen, *ICU* intensive care unit, *MAP* mean arterial pressure, *SOFA* Sequential Organ Failure Assessment

For continuous variables, mean ± standard deviation is given

^a^ *p* = 0.04 for normal liver function vs. mild and severe failure

**Table 2:** Laboratory parameters at start of RCA-CVVHD treatment and end of observation

	Normal liver function (bilirubin ≤2 mg/dl)		Mild liver failure (bilirubin 2–7 mg/dl)		Severe liver failure (bilirubin ≥7 mg/dl)	
	Start	End	Start	End	Start	End
Number of patients	48		43		42	
Total bilirubin (mg/dl)	0.8 ± 0.4	1.1 ± 0.8	3.6 ± 1.3	4.5 ± 3.2	18.4 ± 13.0	17.0 ± 11.2
Aspartate aminotransferase (U/L)	193 ± 454	196 ± 414	716 ± 1864	636 ± 2021	391 ± 1206	391 ± 1336
Alanine aminotransferase (U/L)	154 ± 602	104 ± 161	344 ± 766	361 ± 961	210 ± 476	199 ± 505
Creatinine (mg/dl)	3.5 ± 1.5	1.4 ± 0.7	2.7 ± 1.4	1.4 ± 0.6	2.3 ± 1.1	1.6 ± 1.9
Urea (mg/dl)	123 ± 75	53 ± 24	102 ± 59	49 ± 25	135 ± 69	74 ± 40
Sodium (mmol/L)	141 ± 5	142 ± 4	140 ± 6	142 ± 4	140 ± 7	140 ± 4
Potassium (mmol/L)	4.7 ± 0.7	4.3 ± 0.5	4.6 ± 0.8	4.4 ± 0.6	4.5 ± 0.7	4.3 ± 0.6
Phosphorus (mmol/L)	1.8 ± 0.9	0.8 ± 0.3	1.7 ± 0.8	0.8 ± 0.4	1.5 ± 0.7	1.0 ± 0.5
Magnesium (mmol/L)	1.1 ± 0.4	0.9 ± 0.1	1.0 ± 0.4	0.9 ± 0.2	1.0 ± 0.3	1.0 ± 0.2
Hematocrit (%)	30 ± 4	31. ±4	29 ± 5	30 ± 6	28 ± 5	29 ± 4
Platelet count (count/nl)	195 ± 170	159 ± 164	109 ± 85	92 ± 87	103 ± 91	98 ± 85
INR	1.7 ± 1.0	1.4 ± 0.7	1.5 ± 0.3	1.4 ± 0.4	1.8 ± 0.7	1.7 ± 0.6
aPTT (s)	52 ± 20	46 ± 10	50 ± 12	49 ± 17	59 ± 25	54 ± 16
Serum protein (g/dl)	4.9 ± 1.1	5.2 ± 1.0	4.8 ± 1.1	5.0 ± 1.0	5.2 ± 1.1	5.3 ± 1.2
Serum albumin (g/dl)	2.2 ± 0.6	2.2 ± 0.6	2.1 ± 0.6	2.3 ± 0.9	2.3 ± 0.6	2.3 ± 0.6
Total calcium (mmol/L)	2.0 ± 0.3	2.1 ± 0.2	2.0 ± 0.4	2.3 ± 0.3	2.1 ± 0.3	2.3 ± 0.3
Ratio total/ionized Ca^2+^	1.8 ± 0.1	1.9 ± 0.1	1.8 ± 0.1	1.9 ± 0.2	1.8 ± 0.2	2.0 ± 0.3
Postfilter iCa (mmol/L)	0.27 ± 0.05	0.28 ± 0.03	0.27 ± 0.06	0.29 ± 0.03	0.30 ± 0.03	0.30 ± 0.02

*aPTT* activated partial thromboplastin time, *iCa* ionized calcium, *INR* international normalized ratio

Mean ± SD

### Filter lifetime during RCA-CVVHD

In total, in the group of 133 patients, 181 filters were used. Among all patients, only one filter was used in 69 %, two filters were used in 26 %, and three filters were used in 5 %. In 31 patients, RCA-CVVHD was stopped before 72 h due to recovery and/or transport to another department (n = 22), death (n = 6), or metabolic complications (increase of total calcium [n = 2], metabolic acidosis [n = 1]). The median evaluated treatment time per patient was 71.1 h.

**Fig. 1:**
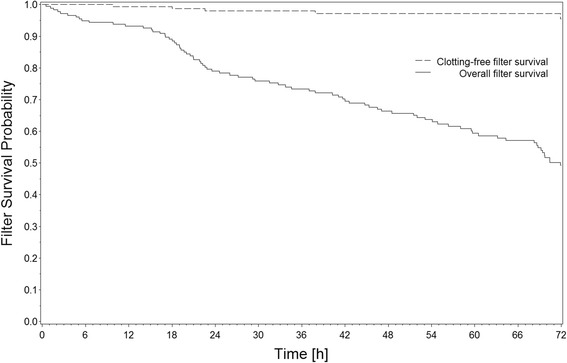
Survival probability of filters of regional citrate anticoagulation and continuous venovenous hemodialysis (RCA-CVVHD) circuits. *Continuous line*: all filter discontinuations. *Dashed line*: Clotting only (i.e., censored for non-clotting events)

Treatment parameters are presented in Table 3. The observation period ended after 72 h. Hence, in cases where the filter was changed within 72 h, the maximum filter lifetime is available for only the first filter. The estimated filter survival for all filters was 56 % at the maximum treatment observation time point of 72 h. The observed median filter lifetime of the first filter without censoring for non-clotting events was 70.4 h. The observed median filter lifetime in the three groups did not differ significantly (normal liver function 70.7 h, mild LF >72 h, severe LF 69.7 h). Overall, only 4 (97.8 %) of 181 filters had to be changed because of clotting before 72 h (Fig. 1). The reasons for discontinuation of treatment were 45 % diagnostic or surgical procedure, 18 % renal recovery or change of therapy, 8 % death, 5 % catheter dysfunction, 15 % CRRT-related, and 9 % other. Among the cases with CRRT-related reasons for discontinuation, five treatments had to be stopped because of a technical malfunction of the CRRT device and four because of filter clotting before 72 h. One additional clotting incident occurred at exactly 72 h. Three treatments were stopped because of metabolic complications (for details, see below). Filter clotting occurred once in the normal liver function group, thrice in the mild LF group, and once in the severe LF group.

**Table 3:** RCA-CVVHD flows and citrate dose at start of treatment and end of observation

	Normal liver function (bilirubin ≤2 mg/dl)		Mild liver failure (bilirubin 2–7 mg/dl)		Severe liver failure (bilirubin ≥7 mg/dl)	
	Start	End	Start	End	Start	End
Blood flow (ml/min)	109 ± 24	114 ± 25	109 ± 25	117 ± 28	110 ± 24	107 ± 20
Dialysate flow (ml/h)	2144 ± 502	2292 ± 500	2128 ± 405	2328 ± 567	2098 ± 403	2319 ± 501
Net ultrafiltration (ml/h)	77 ± 108	74 ± 114	68 ± 100	84 ± 105	84 ± 103	85 ± 102
Citrate dose (mmol/L blood)	4.0 ± 0.1	3.8 ± 0.2	4.0 ± 0.1	3.9 ± 0.3	4.0 ± 0.1	4.1 ± 0.4

*RCA-CVVHD* regional citrate anticoagulation and continuous venovenous hemodialysis

**Fig. 2:**
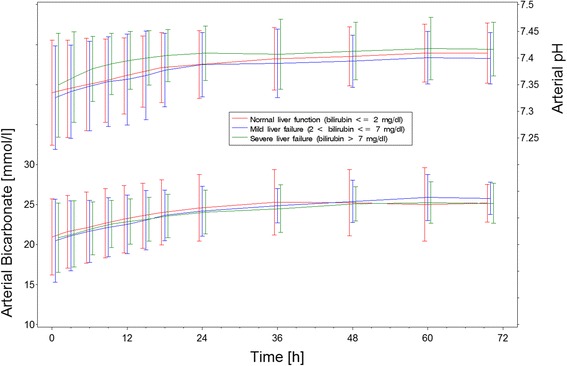
Estimated mean ± standard deviation of arterial pH and serum bicarbonate concentrations during regional citrate anticoagulation and continuous venovenous hemodialysis according to liver groups

**Fig. 3:**
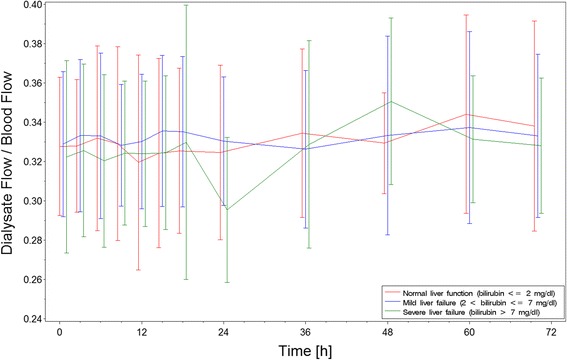
Estimated mean ± standard deviation of blood flow to dialysate flow during regional citrate anticoagulation and continuous venovenous hemodialysis according to liver groups

Mean ± SD. For dialysate to blood flow ratio and calcium dose, see Figs. 2 and 3, respectively

### Episodes of severe acidosis during RCA-CVVHD

A total of 23 new episodes of severe acidosis (pH ≤7.20) of any cause were observed in 19 patients. In seven patients, severe acidosis was present at the initiation of RCA-CVVHD. The minimum arterial pH in all patients with at least one episode of severe acidosis ranged from 6.64 to 7.20, with a median of 7.17. The median time until occurrence or detection of new severe acidosis was 3.9 h (range 0–57.2 h).

Three patients died during the study period without resolving acidosis. These patients were already in critical condition at baseline, with pH ranging from 7.08 to 7.28. One of these patients also developed severe hypocalcemia and is described in detail below (patients with signs of citrate accumulation). Another patient with severe lactic acidosis (pH = 7.0, base excess [BE] = −16.4, lactate = 9.15 mmol/L) was switched after 16 h of RCA-CVVHD to continuous venovenous hemodiafiltration without anticoagulation. All other patients recovered from severe acidosis during RCA-CVVHD within a median of 2 h (range 0.5–25 h).

The frequency distribution of episodes of severe acidosis was not different between the liver function groups (normal liver function 13 %, mild LF 16 %, severe LF 14 %; *p* = 0.95).

### Episodes of severe alkalosis during RCA-CVVHD

Three patients (normal liver function, one patient; mild LF, none; severe LF, two patients; *p* = 0.41) developed four episodes of severe alkalosis (maximum pH 7.56–7.58). No patient had preexisting alkalosis. The time until occurrence of alkalosis ranged from 8.8 to 47.2 h (median 37.2 h). Severe alkalosis resolved in all patients after adjustment (increase) of the dialysate to blood flow ratio within 3.0–6.0 h (median 6 h).

### Acid–base status during RCA-CVVHD

Mean pH and bicarbonate concentration increased gradually during the RCA-CVVHD treatment in all three groups (Table 4, Fig. 2). There was no significant difference in blood pH, bicarbonate, or the blood flow to dialysate flow ratio between the liver function groups (Figs. 2 and 3). The distribution of serum bicarbonate concentrations was not different between groups at study start (*p* = 0.67). The percentages of patients with serum bicarbonate concentration <20 mmol/L were 32 % in the normal liver function group, 44 % in the mild LF group, and 33 % in the severe LF group. Among those patients, the frequency of patients reaching bicarbonate concentrations within normal range (20–26 mmol/L) during treatment and the time until serum bicarbonate concentration reached normal range were similar in all groups (normal liver function 93 %, mild LF 94 %, severe LF 79 %; *p* = 0.42; and normal liver function 13.2 h, mild LF 11.4 h, severe LF 11.8 h; *p* = 0.85). The frequency of serum bicarbonate concentrations >26 mmol/L was comparable (normal liver function 11 %, mild LF 15 %, severe LF 14 %). Of those patients, the frequency of patients reaching bicarbonate concentrations within normal range during treatment and the time until serum bicarbonate concentration reached normal range were similar in all groups (normal liver function 100 %, mild LF 83 %, severe LF 100 %; *p* = 1.0; and normal liver function 17.8 h, mild LF 8.2 h, severe LF 8.0 h; *p* = 0.65).

**Table 4:** Acid–base status at start of RCA-CVVHD treatment and end of observation

	Normal liver function (bilirubin ≤2 mg/dl)		Mild liver failure (bilirubin 2–7 mg/dl)		Severe liver failure (bilirubin ≥7 mg/dl)	
	Start	End	Start	End	Start	End
Arterial pH	7.33 ± 0.10	7.41 ± 0.06	7.33 ± 0.10	7.44 ± 0.05	7.35 ± 0.10	7.42 ± 0.05
Arterial serum bicarbonate (mmol/L)	20.9 ± 4.8	25.2 ± 2.4	20.5 ± 5.2	25.8 ± 2.0	20.8 ± 4.3	24.7 ± 2.7
Base excess (mmol/L)	−4.1 ± 5.8	1.3 ± 3.2	−4.6 ± 6.3	1.4 ± 6.8	−3.9 ± 5.0	−0.9 ± 6.8
Arterial lactate (mmol/L)	2.27 ± 3.61	1.71 ± 2.22	3.78 ± 4.53	2.63 ± 3.25	2.81 ± 2.69	3.41 ± 4.93
Arterial pO_2_ (mmHg)	101 ± 34	96 ± 29	99 ± 31	92 ± 28	93 ± 27	96 ± 21
Arterial pCO_2_ (mmHg)	40 ± 11	41 ± 9	41 ± 11	43 ± 8	39 ± 11	39 ± 7

*pCO*_*2*_ carbon dioxide pressure, *pO*_*2*_ oxygen pressure, *RCA-CVVHD* regional citrate anticoagulation and continuous venovenous hemodialysis

Mean ± standard deviation

### Episodes of severe hypocalcemia during RCA-CVVHD

A total of 15 episodes of severe hypocalcemia (systemic iCa ≤0.9 mmol/L) were observed in 15 patients (median 0.79, range 0.60–0.90 mmol/L). Three patients had preexisting severe hypocalcemia at the beginning of RCA-CVVHD. In five patients, hypocalcemia was detected in the first measurement of iCa during RCA-CVVHD (time 0). In these patients, iCa measurements before the treatment were not available. Therefore, the exact time of onset of hypocalcemia could not be determined, but hypocalcemia was most likely preexistent. In patients with detection or onset of severe hypocalcemia after initiation of RCA-CVVHD, the median time until occurrence was 0.5 h (range 0.0–44.0 h). One patient died during the study period without the state of hypocalcemia resolving. This patient is discussed in detail below. In the other patients, severe hypocalcemia resolved after a median of 18.0 h (range 2.0–36.2 h). In the three patients with preexisting severe hypocalcemia, the time to resolution was 1.4 to 4.9 h. The number of patients with episodes of hypocalcemia according to liver function groups was normal liver function 8 %, mild LF 14 %, and severe LF 12 % (*p* = 0.70).

### Episodes of severe hypercalcemia during RCA-CVVHD

No new severe hypercalcemia (iCa ≥1.5 mmol/L) was observed during RCA-CVVHD. Three patients had preexisting severe hypercalcemia. All of these cases resolved, two within the first 30 minutes of RCA-CVVHD treatment.

### Systemic iCa and calcium dose during RCA-CVVHD by group

**Fig. 4:**
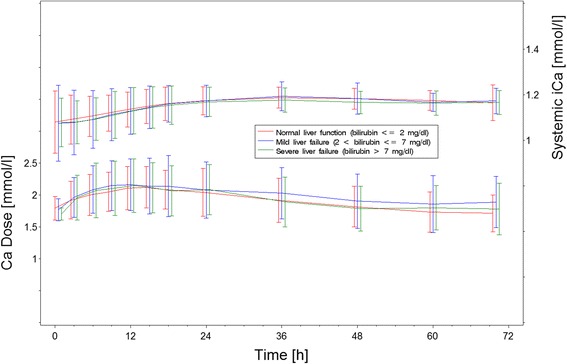
Estimated mean ± standard deviation of systemic arterial ionized calcium (iCa) and calcium dose during regional citrate anticoagulation and continuous venovenous hemodialysis according to group

As shown in Fig. 4, calcium dose and systemic iCa values did not differ between the study groups (*p* = 0.42 for calcium dose and *p* = 0.85 for iCa).

### Bleeding episodes

Three patients (2 %) bled at the initiation of RCA-CVVHD, and another five developed bleeding during RCA-CVVHD (4 %).

### Citrate dose during RCA-CVVHD

The mean ± SD citrate dose at the start of the study was 4.0 ± 0.1 mmol/L in each study group, and doses were not different after 70 h of RCA-CVVHD (normal liver function 3.8 ± 0.2, mild LF 3.9 ± 0.3, severe LF 4.1 ± 0.4; *p* = 0.17). Furthermore, there was no relationship between INR and the difference between citrate dose at the start of the study and after 70 h (Spearman’s ρ = 0.03), indicating that there was no detectable intention or need to reduce the citrate dose in patients with higher INRs.

### Patients with signs of citrate accumulation

Overall, three patients showed signs of citrate accumulation [11]. In three patients, the ratio of systemic total calcium to iCa increased ≥2.5, and one of them also had increased total calcium (≥2.8 mmol/L).

The first of these patients was admitted to the ICU with fulminant acute LF and MODS (bilirubin 16.1 mg/dl, INR 2.0) related to graft-versus-host disease after stem cell transplantation. Lactic acidosis (pH = 7.28, oxygen pressure = 66 mmHg, carbon dioxide pressure = 45 mmHg, BE = −5.3 mmol/L, lactate = 3.55 mmol/L) was present at the start of RCA-CVVHD. The patient died as a result of refractory shock in severe lactic acidosis (pH = 7.06, BE = −23.4, lactate = 23.3 mmol/L) after 47 h of treatment. This patient’s last systemic iCa measurement was 0.90 mmol/L and last total calcium level was 2.31 mmol/L. Thus, the ratio of total calcium to iCa was not severely increased (i.e., 2.57).

The second patient was admitted with severe sepsis and MODS after partial liver resection (bilirubin 18.8 mg/dl, INR 1.2). Serum chemistry at the start of the RCA-CVVHD showed hyperlactatemia (2.5 mmol/L) and normal calcium status. A slight decrease of iCa was observed after 15 minutes of RCA-CVVHD treatment, and iCa decreased continuously, reaching a nadir of 0.85 mmol/L after 12 h (total calcium 2.76 mmol/L, total calcium/iCa ratio 3.24). Calcium substitution was increased in stepwise fashion to 2.7 mmol/L, and the citrate dose was likewise decreased to 2.5 mmol/L. After the next 15 h, both iCa and total calcium totally returned into the normal range, and the corresponding ratio was 2.07.

The third patient was admitted to the ICU with fulminant acute LF due to acetaminophen intoxication (bilirubin 11.6 mg/dl, INR 4.8). The first iCa level, measured 40 minutes after initiation of RCA-CVVHD, was 0.9 mmol/L, and the calcium dose was increased from 1.7 to 2.0 mmol/L. After 6 h, this patient’s iCa level decreased further to 0.62 mmol/L (i.e., severe hypocalcemia was present). The calcium dose was further increased to 3.0 mmol/L, and the citrate dose was decreased to 3.0 mmol/L. Thereafter, the patient’s iCa concentration gradually increased and reached normal range after 16 h. With these treatment settings, iCa remained stable but total calcium concentration increased to 3.72 mmol/L (total calcium/iCa ratio 3.23), indicating citrate accumulation [2]. There were no significant changes in the patient’s acid–base parameters, and the patient’s INR was 1.9 at this time point. Nevertheless, ongoing citrate accumulation was suspected, and RCA-CVVHD was stopped after 41 h.

## Discussion

To our knowledge, this is the first multicenter prospective study on the efficacy and safety of a citrate-based CVVHD protocol in patients with liver dysfunction. The major results are, first, that RCA can be applied safely and effectively in patients with severely impaired liver function and, second, that in these patients filter running times with RCA are extraordinarily long. A great number of patients with different degrees of LF in medical and surgical ICUs were recruited. This allowed us to conduct comprehensive analyses based on a heterogeneous study population, and the database is sufficiently large to support the conclusions.

In the present study, filter clotting was a very rare event. The filter lifetime censored for non-clotting treatment interruptions even improved compared with the first results published some years ago [6], using the same protocol for RCA-CVVHD. In that early publication, filter survival with censoring for non-clotting events was 63 % at 72 h. In the present study, it was 98 %. This improvement can be explained by technical improvements of RCA, such as the integration of citrate and calcium pumps into the CRRT device and partial automation of dose adaptations for citrate and calcium. However, the present study shows, for the first time to our knowledge, that with this protocol liver dysfunction has no effect on the incidence of filter clotting. In addition, experience in the use of RCA has increased.

Most important, there were no differences in the ability to metabolize citrate in critically ill patients with liver dysfunction compared with those with normal liver function. There was no increased risk for acidosis and no need to lower the dialysate to blood flow ratio to counteract metabolic acidosis as one would expect in cases of insufficient metabolism of citrate to bicarbonate (Fig. 3). This finding is in line with a prospective observational study in patients with severe LF treated with RCA-CVVHD [15]. In another observational study, patients treated with an extracorporeal liver support system also tolerated RCA [16]. Of note, in the present study, acidosis resolved in 129 of 133 patients within the 72-h observation period. Persistent acidosis is a well-known indicator of high mortality; in the present study, three of the patients with persistent acidosis died. The incidence of severe alkalosis was also independent of liver dysfunction. Alkalosis per se was mild, and its overall incidence was even lower than reported for standard bicarbonate-buffered modalities of CRRT with systemic anticoagulation [7, 17]. With the use of an algorithm-guided adaption (increase) of the dialysate to blood flow ratio, all alkalosis episodes resolved within at most 6 h, showing again that RCA-CVVHD allows rapid correction of metabolic alkalosis [10].

Seven patients developed severe hypocalcemia during the study period. Again, the incidence was independent of liver dysfunction. Because the number of patients and especially the number of events were small, the observed difference was not statistically significant, and there is a probability of a type II error. No increase in the need for calcium supplementation was observed in patients with liver dysfunction, as could have been expected in cases of impaired citrate metabolism and thus lack of release of iCa from calcium-citrate complexes. Surprisingly, the incidence and severity of hypocalcemia is similar to that in other studies on the incidence of hypocalcemia in critically ill patients without renal replacement therapy [18, 19]. No new episodes of severe hypercalcemia occurred during RCA-CVVHD treatment. Preexisting hypercalcemia was treated with RCA-CVVHD because it allows control of arterial iCa in both directions by adapting calcium supplementation [20].

Signs of citrate accumulation were observed in three patients. In one patient, the citrate accumulation was only mild and transient. The second patient had concomitant severe lactic acidosis. The most severe citrate accumulation was found in the third patient, who had acetaminophen intoxication. Citrate is metabolized via the citric acid cycle, and its metabolism is oxygen-dependent [21–23]. A state of intracellular hypoxemia or an otherwise inhibited cellular respiration leads to impaired citrate metabolism. Hyperlactatemia and lactic acidosis are signs of an anaerobic metabolic state. This finding is in line with a retrospective analysis in patients treated with RCA-CVVHD, showing that severe lactic acidosis is strongly associated with impaired citrate metabolism [24]. Interestingly, the mechanism of action in acetaminophen intoxication is inhibition of mitochondrial respiration [25, 26]. We hypothesize that it is not the state of liver function per se but a sufficient cellular respiration that is essential for metabolism of citrate. Median lactate concentrations were low and did not differ significantly between the liver function groups. This is the most likely reason why we did not find a difference in the ability to metabolize citrate between the groups. Liver dysfunction alone is not the major cause of citrate accumulation.

In the present study, citrate dose was not different in patients with LF. The relationship of coagulatory activity and blood iCa concentration is not linear; that is, the thromboelastography is totally unaffected with iCa concentrations >0.56 mmol/L [27, 28]. As shown before, for an effective anticoagulation of the extracorporeal circuit, a target range of 0.25–0.35 mmol/L for iCa is required. In cases of normal arterial iCa, a dose of approximately 4 mmol of citrate per liter of blood is required to lower iCa into this target range. The present study shows that these recommendations are appropriate in patients with LF.

An apparent limitation of this study is the choice of serum bilirubin as a parameter for liver function. Because no physiologic variable available for clinical use allows early detection of hepatic dysfunction, current diagnostic criteria are based on laboratory tests, mostly serum bilirubin. Unlike other diagnostic criteria, serum bilirubin remains a stable and powerful marker of hepatic dysfunction, with elevated levels reflecting impairment in the energy-consuming processes of heme metabolism, conjugation, and bile secretion (for review, see [29]). One may argue that a parameter of liver synthesis and function—namely, INR and Model for End-Stage Liver Disease (MELD) score—may better correlate with the ability of the liver to metabolize citrate. However, INR increases often do not occur early in LF and represent only severe forms of LF. Furthermore, INR is also increased in patients with disseminated intravascular coagulation frequently observed in severe sepsis, and it is influenced by substitution of plasma products. The MELD score is approved for assessing end-stage chronic liver disease and not for acute hepatic dysfunction, which was the presentation of the majority of the patients in our study population.

Serum bilirubin is a key component of prognostic models in patients with acute LF [30]. Hence, we decided to use bilirubin for classification of liver function in the present study. This view is shared by others. Meier-Kriesche et al. [11] used bilirubin for classification of liver dysfunction in a study of RCA for CVVHD. They defined LF as serum bilirubin >7 mg/dl. Even though citrate load in their protocol was comparable to ours (9.3 g/h vs. approximately 11 g/h), they reported a much higher incidence of citrate accumulation as we observed in our present study, using the same definition as we did—namely, an increase in the total calcium to iCa ratio ≥2.5. The mortality of the patients with citrate accumulation in their study was very high, as 17 of 19 patients died during the hospital stay. One can assume that the unfavorable outcome of severely ill patients with LF and citrate accumulation in the study of Meier-Kriesche et al. is in line with impaired cellular respiration, as it was found to be the major cause of disturbed citrate metabolism in the present study. Furthermore, the 12 % incidence of citrate accumulation in the total cohort treated with RCA was rather high compared with other studies on RCA, with incidences of 0–1.24 % [3, 4, 6, 7], and with the present study (2.3 %). In the present study, the majority of patients showed LF secondary to ischemia or multiple organ failure and not to primary liver disease as could be expected in a mixed ICU patient population. On the basis of our data, a higher incidence of citrate accumulation in patients with LF due to primary liver disease cannot be excluded. Another issue might be seen in the limitation of the observation period to 72 h of treatment. According to the citrate metabolism kinetics, the increase in the systemic citrate concentration responsible for citrate accumulation can be expected to occur quickly in the first hours of treatment, whereas afterward a slower increase, or even a plateau phase, should be achieved [31]. In that regard, if the patient is at risk of accumulation of citrate, this accumulation should be noticeable shortly after treatment start. A 72-h observation period seems to be reasonable because the patients in the liver dysfunction groups were at risk for citrate accumulation from the beginning of treatment.

## Conclusions

The investigated protocol for RCA-CVVHD can be used safely in patients with liver dysfunction. Furthermore, it yields excellent filter patency and thus can be recommended as first-line anticoagulation in almost all ICU patients. Caution must be taken in patients with impaired cellular respiration.

## Key messages

- This citrate anticoagulation protocol for CVVHD can be used safely in patients with LF.
- It yields excellent filter patency and thus can be recommended as first-line anticoagulation.
- Citrate accumulation in patients with LF is rare, but caution is needed, particularly in patients with impaired cellular respiration.

## Abbreviations

AKI: Acute kidney injury
aPTT: Activated partial thromboplastin time
BE: Base excess
CRRT: Continuous renal replacement therapy
FiO_2_: Fraction of inspired oxygen
iCa: Ionized calcium
ICU: Intensive care unit
INR: International normalized ratio
LF: Liver failure
MAP: Mean arterial pressure
MELD: Model for End-Stage Liver Disease
MODS: Multiple organ dysfunction syndrome
OUS: Oslo University Hospital, Norway
pCO_2_: carbon dioxide pressure
pO_2_: oxygen pressure
RCA: Regional citrate anticoagulation
RCA-CVVHD: Regional citrate anticoagulated continuous venovenous hemodialysis
RIFLE: Risk, injury, failure, loss of kidney function, end-stage kidney disease
RRT: Renal replacement therapy
SD: Standard deviation
SOFA: Sequential Organ Failure Assessment
